# Evaluation of testicular glycogen storage, FGF21 and LDH expression and physiological parameters of sperm in hyperglycemic rats treated with hydroalcoholic extract of *Securigera Securidaca* seeds, and Glibenclamide

**DOI:** 10.1186/s12958-021-00794-1

**Published:** 2021-07-07

**Authors:** Mohammad Babaei, Shahin Alizadeh-Fanalou, Alireza Nourian, Sahar Yarahmadi, Navid Farahmandian, Mohsen Nabi-Afjadi, Iraj Alipourfard, Elham Bahreini

**Affiliations:** 1 Department of Clinical Sciences, Faculty of V, eterinary Science, Bu-Ali Sina University, Hamedan, Iran; 2 Department of Biochemistry, Faculty of Medicine, Iran University of Medical Sciences, Tehran, Iran; 3 Department of Pathobiology, Faculty of Veterinary Science, Bu-Ali Sina University, Hamedan, Iran; 4 Department of Biochemistry, Faculty of Biological Science, Tarbiat Modares University, Tehran, Iran; 5 Institute of Biology, Biotechnology and Environmental Protection, Faculty of Natural Sciences, University of Silesia, Bankowa 9, 40-007 Katowice, Poland

**Keywords:** *Securigera Securidaca*, Glibenclamide, Sperm parameters, Fertility, FGF21, LDH, Glycogen

## Abstract

Structural and physiological changes in sperm and semen parameters reduce fertility in diabetic patients. *Securigera Securidaca* (*S. Securidaca*) seed is a herbal medicine with hypoglycemic, antioxidant, and anti-hypertensive effects. The question now is whether this herbal medicine improves fertility in diabetic males. The study aimed to evaluate the effects of hydroalcoholic extract of S. Securidaca seeds (HESS), glibenclamide and a combination of both on fertility in hyperglycemic rats by comparing histological and some biochemical changes in testicular tissue and sperm parameters. The treatment protocol included administration of three doses of HESS and one dose of glibenclamide, as well as treatment with both in diabetic Wistar diabetic rats and comparison of the results with untrated groups. The quality of the testicular tissue as well as histometric parameters and spermatogenesis indices were evaluated during histopathological examination. Epididymal sperm analysis including sperm motility, viability, abnormalities, maturity, and chromatin structure were studied. The effect of HESS on the expression of LDH and FGF21 genes and tissue levels of glycogen, lactate, and total antioxidant capacity in testicular tissue was investigated and compared with glibenclamide. HESS improved sperm parameters in diabetic rats but showed little restorative effect on damaged testicular tissue. In this regard, glibenclamide was more effective than the highest dose of HESS and its combination with HESS enhanced its effectiveness so that histological tissue characteristics and sperm parameters were were comparable to those of healthy rats. The expression level of testicular FGF21 gene increased in diabetic rats, which intensified after treatment with HESS as well as glibenclamide. The combination of HESS and glibenclamide restored the expression level of testicular LDH gene, as well as tissue storage of glycogen, lactate and LDH activity, and serum testosterone to the levels near healthy control. S. Securidaca seeds can be considered as an effective supplement in combination with hypoglycemic drugs to prevent infertility complications in diabetes.

## Introduction

A proven feature of diabetes mellitus (DM) is the reduction in male fertility, both in terms of semen parameters and sperm structures [1, 2]. These effects are described as devastating changes in spermatogenesis and testicular tissue through pre-testicular, testicular, and post-testicular mechanisms. Pre-testicular failure mostly occurs in the hypothalamic-pituitary–gonadal axis and results in testosterone deficiency [3, 4]. The testicular mechanism induced by elevated oxidative stress and non-enzymatic glycation products (AGEs) [5], sperm DNA fragmentation [6], and mitochondrial bioenergy alteration [7]. Moreover, the most common post-testicular cause is infection and inflammation of the male accessory gland [8, 9]. The process of spermatogenesis is strictly regulated by the function of Sertoli cells, which maintain the division of germ cells and provide supporting factors such as hormones, various pro-, and anti-apoptotic agents, and energetic substrates. Sertoli cells metabolize glucose to pyruvate through cytoplasmic glycolysis, immediately reduce it to lactate by lactate dehydrogenase (LDH), and export it to the intratubular fluid by monocarboxylate transporter 4 (MCT4). Lactate is the preferred energy source for the development of postmeiotic germ cells, especially spermatocytes and spermatids. The released lactate is taken up by germ cells via MCTs and converted to pyruvate, while the resulting NADH can modify the redox status of germ cells. This metabolic pattern is referred to as “Warburg-like metabolism” and is frequently observed in cancer cells [10–12]. Galardo et al. [13] showed that lactate increased mRNA levels of MCT2 and LDH-C, but not MCT4 and LDH-A.

MCTs, are a family of proton-linked plasma membrane transporters that carry monocarboxylates across biological membranes. MCT2 is a transporter predominantly expressed in germ cells with a high affinity for lactate [14], while MCT4 is with lower affinity one and responsible for sending out lactate of highly active glycolytic cells such as Sertoli cells [15]. LDH isozymes are tetramers composed from the two most common subunits, of LDH-M and LDH-H protein, that are encoded by the *LDHA* and *LDHB* genes, respectively. However, there are two more mammalian LDH subunits that can be included in LDH tetramers: LDHC and LDHBx. LDH-C is a testes-specific LDH protein, that is encoded by the *LDHC* gene. It is found only in germ cells and preferentially converts lactate to pyruvate. In contrast, LDH-A preferentially converts pyruvate to lactate and is more likely to be expressed in Sertoli cells, which produce large amounts of lactate [16, 17]. Since lactate is the primary source of ATP and sperm activity, any failure of glucose uptake, lactate transport, and the LDH-C gene can disrupt sperm motility patterns and male fertility [16]. The presence of glycogen and enzymes involved in its metabolism in testicular tissue has already been reported. In abnormal sufficiency of insulin and glucose concentration, testicular glycogen is an alternative fuel [18] which is documented by increase in uridine diphosphate (UDP) glucose as the glycosyl donor for glycogen synthesis [19].

Fibroblast growth factor 21 (FGF21), as an important metabolic regulator produced mainly in liver as well as in skeletal muscle, adipose tissue and to a lesser extent in in testis, plays a key role in glucose homeostasis, insulin sensitivity, and ketogenesis, as well as normal spermatogenesis [20]. FGF21 maintains normal germ cell apoptosis and spermatogenesis homeostasis by suppressing p53 activation via MDM2 (human murine double minute 2) through AKT, especially AKT1 [21]. Although high blood sugar and oxidative stress products stimulate FGF21 expression, deficiency of FGF21 enhances germ cell apoptosis due to diabetes -induced oxidative damage [20].

The plant *S. securidaca* (L.) Degen & Dorfl (Bitter Lentils) is widely used as a medicinal plant in traditional Iranian medicine locally known as names Adasol-Molk and Gande-Talkhe. Egyptians and Indians used it to treat high blood sugar in patients. In support of the chemical analysis, Garani et al. showed the presence of flavonoids, saponins, tannins and alkaloids in the hydroalcoholic extract of *S. Securidaca* [22]. The further phytochemical analysis by Aldal'in et al. showed high content of aromatic derivatives, dodecanedioic acid derivatives and L-ascorbic acid, and β-sitosterol and oxygenated hydrocarbons such as, Acyl Glucuronides, α-D-glucopyranose, N-butylglycine and 1, 3-propanediol in this plant [23]. The plant has been used for the treatment of hyperlipidemia, hypertension and as also antioxidant. The hydroalcoholic extract of *S. securidaca* seeds (HESS) has been shown to have facilitative effects as an adjunct to classic diabetes medications such as glibenclamide [24, 25]. In the current study, the impact of HESS, glibenclamide and the combination of both on the testes of the diabetic rat were investigated by comparing histological and some biochemical alterations in testicular tissue and changes in sperm parameters. To our knowledge, this is the first study to evaluate the effects of HESS and glibenclamide separately and in combination on testicular tissue and sperm parameters in hyperglycemic male rats.

## Materials and methods

### The preparation of hydroalcoholic seed extract and estimation of total phenolic and flavonoid content

The hydroalcoholic extract of the *S. securidaca* seed (herbarium code of PMP-756) and measurement of the total phenolic and flavonoid contents have been fully described in the previous article [24]. Briefly, the ground seed was extracted using 70% ethanol and concentrated by rotary evaporation. According to the method described by Singleton and Rossi, Folin Ciocalteu reagent and gallic acid were used as standard to measure the total phenolic content [26]. In addition, the flavonoid content of the extract was determined by the aluminum chloride colorimetric method and quercetin was used as the standard [27].

### Experimental animals

The sample size calculation was done based on the resource equation [28]. Forty eight healthy male Wistar rats with an average weight of 240 g were obtained from Laboratory Animal Center of Iran University of Medical Sciences, and kept in a well-ventilated room with free access to food and water for one week before the experiments. Animals were randomly allocated to 8 groups of 6 rats. After isolating 6 mice as a healthy control group (without treatment), hyperglycemia was induced in the rest of the animals by intraperitoneal injection of Streptozotocin (STZ, 55 mg/kg-BW). A blood sugar level of ≥ 200 mg/dL was considered as diabetic. The animal work was performed according to the ethical considerations of working with laboratory animals approved by the Ministry of Health of Iran and the instructions of the laboratory animal care department of Iran University of Medical Sciences. (https://ethics.research.ac.ir/docs/Ethics-Lab-Animal-Codes.pdf).

#### Experimental design

Three doses of HESS (100, 200, and 400 mg/kg-BW) [29] and one dose of glibenclamide (5 mg/kg-BW) were considered. The experimental groups were as follows: (I) normal (healthy) control (NC), (II) diabetic control (DC), (III) to (V) diabetic rats treated with HESS doses of 100, 200 and 400 mg/kg-BW as E-100, E-200 and E-400 groups, respectively, (VI) diabetic rats treated with 5 mg/kg-BW glibenclamide (G) [30], and groups (VII) and (VIII) treated with both glibenclamide and HESS (200 and 400 mg/kg-BW) as G + HESS-200 and G + HESS-400 groups, respectively. Administration of HESS and glibenclamide was carried out by gavage once a day for 35 days.

### Sampling

At the end of the study, the rats were anesthetized with chloroform. The blood sample was collected through cardiac puncture and centrifuged (2000 g, 10 min, 23 °C). The resultant serum was separated and stored at -20 °C until analysis. Immediately after sacrificing the animals, the testes were removed and their size, weight, and appearance were evaluated by an experienced person. Left testicles were placed in 10% neutral buffered formalin for histological evaluation and the right ones were used for molecular and biochemical examination. The gonadosomatic indices (GSI), as an indicator of gonadal development or sexual maturity of an animal, were calculated as follows: (testes weight/body weight) × 100 [31].

#### Epididymal sperm analysis

The caudal region of the epididymis was cut into small segments, which were added to 1 mL human tubal fluid (HTF) containing 4 mg bovine serum albumin (BSA). The container was then incubated for 10 min at 37 ºC with 5% CO2 to let the sperm to swim out of tubules. Epididymal spermatozoa were counted by a hemocytometer according to the standard hemocytometric method. The epididymal sperm was diluted at 1:20 in HTF solution, and 10 μL of the suspension was transferred to the counting chamber of the hemocytometer. After resting for 5 min in a humid chamber for sedimentation of the cells and prevention from drying, the spermatozoa were counted by a light microscope at 400 × magnification and considered as the number of cells per mL.

- **The sperm motility** was examined using an optical microscope at a visual magnification of 400 × . 20 μL of sperm suspension was placed on a microscope slide and a coverslip was placed on it. The quantity of sperm motility was evaluated in 10 microscopic fields, and was expressed as the percentage of motile sperms.
- **The sperm viability** was determined by Eosin-Y (0.05%) [32]. Twenty µL of sperm suspension was added into 20 µL Eosin-Y and the mixture was incubated at room temperature for 2 min. Then, the viability of sperm was assessed microscopically (400 ×). In this method, the spermatozoa with intact plasma membrane are not stained, while the cells with a modified plasma membrane turn pink. The viability was estimated by the ratio of “unstained /pink spermatozoa “and reported as a percentage.
- **The sperm abnormalities** were evaluated by preparation of the sperm smears on clean slides. After air-drying, the slides were stained by Eosin-Y (1%)-Nigrosine (5%). Morphological abnormalities such as amorphous, hook-less, bi-cephalic, coiled, or abnormal tails and attached cytoplasmic droplets were analyzed using a microscope (400 ×) and reported percentage.
- **Evaluation of the sperm chromatin structure** was carried out by the Acridine orange test by slight modifications [33], which indicates the extent of chromatin denaturation. The air-dried smears of the sperm suspension on glass slides were fixed for 2 h in methanol-acetic acid (1:3, v/v). The slides were stained with Acridine orange solution (19%) in phosphate citrate buffer for 5 min, washed with deionized water, and visualized by a fluorescence microscope. The spermatozoa with intact chromatin structure exhibited a green fluorescence while those with denatured chromatin showed a reddish-orange color.
- **The sperm maturity** was evaluated by Aniline blue staining by slight modifications [34], which differentiates lysine-rich histone proteins in immature and arginine/cysteine-rich protamine in mature sperm nucleus. The values were reported as a percentage.

### Biochemical Assay

The effects of various doses of HESS, alone and in combination with glibenclamide, on body weight, cardiovascular indices and some serum biochemical parameters as well as oxidant and antioxidant factors were assessed in our previous studies [24, 25]. In this study, serum testosterone and testicular tissue concentration of LDH, glycogen and total antioxidant were determined as:

**-Testosterone:** the circulating levels of testosterone were measured using a radioimmunoassay kit (Rat Testosterone Kit, Mybiosurce) according to the manufacturer instructions. The assay sensitivity was 0.04 ng/mL.
**-Tissue levels of lactate and LDH activity:** After rinsing with 0.01 M PBS buffer, the right testicles of all groups were cut sagittally to right and left halves for molecular and biochemical studies, respectively. After weighing the samples in cold conditions, the testicular tissue washomogenized in 0.1 M cold PBS (pH 7.0) containing EDTA using a rotary homogenizer. The homogenate was centrifuged at 3000 g for 10 min at 4 °C and the supernatant was kept at − 80 °C until analyzed.
-Total protein concentrations were determined by the Lowry method. Tissue levels of lactate and LDH were measured using Rat ELISA Kits (MBS269777, MyBioSource, USA) according to the manufacturer's instructions. The levels of lactate and LDH were reported as nm/mg-tissue and U/mg- total protein-tissue (specific activity), respectively.
**-Testicular glycogen:** The amount of glycogen in tissues was determined by aRat Glycogen (GLY) ELISA kit (MBS729293, MyBioSource, USA) and was reported as µg/mg-tissue.
**-Total tissue antioxidant levels:** The total antioxidant level of testicular tissue was measured by ferric reducing antioxidant power (FRAP) [35]. The reduction of Fe^3+^ to Fe^2+^ by the sample was considered as an antioxidant power indicator. In this method, the complex between Fe^2+^ and tripyridyltriazine (Fe^2+^-TPTZ) creates a blue color with maximum light absorption at 593 nm. The results were compared with the standard curve derived based on a serial dilution of Fe_2_SO_4_ (ranging from 100 to 800 μMol) in 1 mL of FRAP reagent (300 mMol acetate buffer, 10 mMol TPTZ/HCl solution, and 20 mMol ferric chloride). The values were expressed as µMol Fe^2+^/g-tissue weight.

### Total RNA extraction, cDNA synthesis and Quantitative Real-Time PCR (RT-PCR)

The study of gene expression levels of testicular LDH and fibroblast growth factor-21 (FGF-21) was performed by Quantitative Real-Time PCR (RT-qPCR) technique. About 20–30 mg testicular tissue was frozen in liquid nitrogen and pulverized in a mortar and pestle in more liquid nitrogen. The powdered tissue was homogenized in TRIzol Reagent and total RNA was extracted using Total RNA Extraction kit (cat. no. FAPDE050, Yektatajhize, Iran), according to the manufacturer's protocol. After quantitative and qualitative evaluation of the purified RNA using a Nanodrop (ND-1000 spectrophotometer, Yektatajhize, Iran), the total RNA was reverse-transcribed into first-strand complementary DNA (cDNA) using cDNA Synthesis Kit (cat. no. YT4500, Yektatajhize, Iran) according to the manufacturer's instruction. The GAPDH (glyceraldehyde 3-phosphate dehydrogenase) gene was used as an internal control to quantify the expression of the target genes. Two target genes (LDH and FGF-21) and GAPDH internal control were amplified with suitable primers designed by NCBI primer design (Table 1). The synthesized cDNA was subjected to RT-qPCR quantitation using Super SYBR Green qPCR MasterMix (cat. no. YT2552, Yektatajhize, Iran) in a 20 μL reaction mixture containing RT-PCR Master Mix (10 μL), forward and reverse primers (1 μL), double distilled H_2_O (6 μL) and cDNA (3 μL). The initial denaturation was 95 °C for 3 min, 40 cycles of denaturation at 95 °C for 10 s, annealing at 60 °C for 10 s, elongation at 72 °C for 30 s, and final elongation at 72 °C for 5 min. The cycle threshold (Ct) of amplification was determined and normalized by the value of GAPDH. The expression fold change was calculated using the 2^−ΔΔCt^ method.

**Table 1:** Primer sequences for RT-qPCR

**Gene**	Forward primer (5′-3′) Reversed primer (5′-3′)	Melting temperature (Tm)
GAPDH	TCATCAACGGCACAGTCAAGG TTCTGCATGGTGGTGAAGACG	60.88 60.88
LDH-C	TTTAGCCTTTTCCTCAGCACTCC AGTCTAGGTTACAGCCACTTCC	60.81 59.17
FGF-21	TTCGGGACTGTGGGTCTGTCTC TTTGCAGGTGGGCTTCGGTG	62.6 62.1

### Histopathology

After fixation in neutral buffered formalin, the testicles were dehydrated in ascending ethanol concentrations, cleared in xylene, and infiltrated and embedded in paraffin. Then, using a rotary microtome (Leica RM2255, Germany), the tissue blocks were sectioned at 5–6 µm, stained with hematoxylin and eosin (H&E), and examined independently by a veterinary pathologist using a light microscope (Olympus CX41, Japan) equipped with a digital camera (Olympus DP25, Germany).

**Fig. 1:**
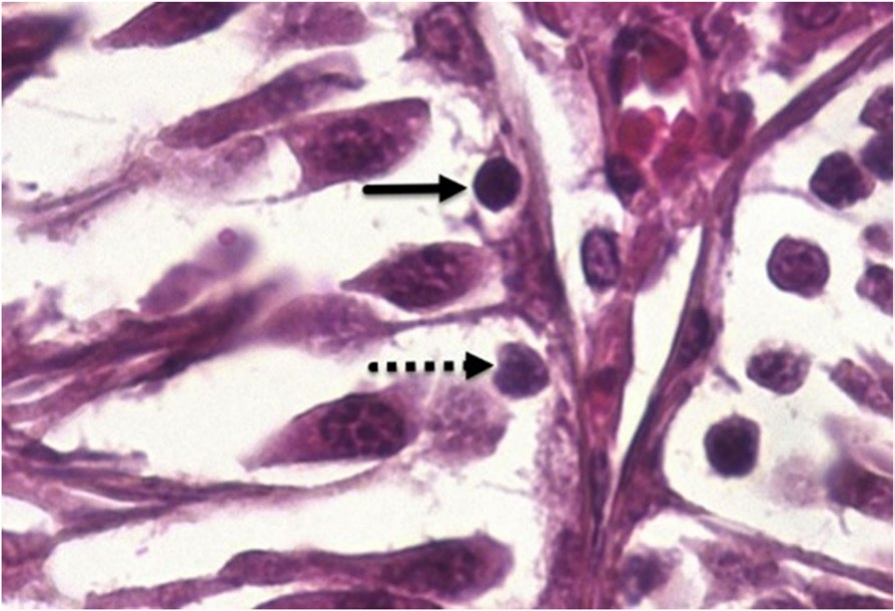
Type **A** spermatogonia (dotted arrow): inactive type with pale nuclei; Type **B** spermatogonia (arrow): the active type with dark nuclei

Spermatogenesis indices were investigated through tubular differentiation index (TDI) and repopulation index (RI) and spermatogenesis index (SI). For measuring TDI, SI and RI, one transverse section was taken from the center of one testis from each rat, and at least 200 seminiferous tubule cross sections were scored in that section. The TDI is the percentage of seminiferous tubules that contained three or more differentiated layers of germinal cells derived from type A spermatogonia. To find out the RI, the ratio of active (type B) to inactive (type A) spermatogonia (Fig. 1) was recorded and SI was expressed as the ratio of the number of seminiferous tubules with spermatozoids to empty tubules.

The histometrical parameters including seminiferous tubules diameter (STsD), germinal epithelium height (GEH), testicular capsule diameter (TCD), seminiferous tubules lumen diameter (STsLD) and Tubular degeneration percentage (TDP) were evaluated by microscopical examination of sections. The Histometrical studies were carried out using Dino-Lite lens digital camera and Dino-capture 2 Software. The STsD, GEH, TCD, and STsLD were expressed as mean micrometer and the TDP was the percentage of degenerated tubules.

### Statistical analysis

The statistical analyses were performed using SPSS software version 22 (SPSS Inc., USA). The Shapiro–Wilk normality test was used to verify the normality of the data. Considering the normal distribution of the data, parametric tests were used. Differences among individual groups were determined by one-way ANOVA analysis followed by Tukey’s test for pair-wise comparisons. The values were expressed as the means ± standard deviation (SD). A *p* value of < 0.05 was considered statistically significant.

## Results

The results of epididymal sperm analysis from all studied groups have been summarized in Table 2.

**Table 2:** Effects of different doses of HESS and glibenclamide on gonadal mass and sperm parameters

Groups	TW (g) (single testis)	GSI (%)	Total sperm count (× 10^7^) /mLof HTF	Viability (%)	Motility (%)	DNA-damage (%)	Abnormality (%)	Maturity
NC	1.62 ± 0.09^b^	0.45 ± 0.05	99.40 ± 2.7 ^b^	89.4 ± 3.6 ^b^	87.8 ± 3.1 ^b^	6.4 ± 1.8 ^b^	11.6 ± 1.8 ^b^	98.4 ± 1.5 ^b^
DC	1.33 ± 0.05^a^	0.55 ± 0.09	48.60 ± 3.5 ^a^	47.8 ± 5.8 ^a^	54.0 ± 6.0 ^a^	34.2 ± 2.6 ^a^	38.0 ± 3.4 ^a^	84.8 ± 0.5 ^a^
HESS-100	1.44 ± 0.03^a^	0.53 ± 0.04	50.40 ± 3.6 ^a^	58.4 ± 2.9 ^a^	56.6 ± 6.6 ^a^	30.4 ± 1.1 ^a^	34.0 ± 4.5 ^a^	86.4 ± 2.1 ^a^
HESS-200	1.47 ± 0.07	0.52 ± 0.06	57.80 ± 4.2 ^a^	63.4 ± 4.0 ^a^	60.8 ± 4.8 ^a^	28.8 ± 1.6 ^a^	30.4 ± 5.6 ^a^	87.0 ± 1.2 ^a^
HESS-400	1.52 ± 0.1^b^	0.53 ± 0.01	60.50 ± 3.1 ^a^	71.0 ± 2.9 ^a^	63.0 ± 5.1 ^a^	24.7 ± 1.3 ^a^	28.0 ± 1.8 ^a^	90.2 ± 1.7 ^a^
G	1.54 ± 0.1^b^	0.57 ± 0.06	94.20 ± 2.4^b^	80.0 ± 3.8 ^b^	79.2 ± 4.1 ^b^	11.6 ± 2.4 ^b^	17.2 ± 2.9 ^b^	94.4 ± 2.7 ^b^
G + HESS-200	1.49 ± 0.04^b^	0.54 ± 0.06	96.80 ± 3.5 ^b^	85.6 ± 4.8 ^b^	82.4 ± 2.4 ^b^	11.0 ± 2.7 ^b^	13.0 ± 3.1 ^b^	95.0 ± 1.6 ^b^
G + HESS-400	1.50 ± 0.05^b^	0.52 ± 0.06	98.80 ± 1.9 ^b^	86.2 ± 4.3 ^b^	85.4 ± 4.6 ^b^	10.6 ± 2.9 ^b^	11.8 ± 4.1 ^b^	97.0 ± 2.7 ^b^
P-value	0.001	0.93	0.001	0.001	0.001	0.001	0.001	0.001

Testicular weight (TW), gonadosomatic indices (GSI), HESS (hydroalcoholic extract of *S. securidaca* seeds); G (glibenclamide); DC (diabetic control); NC (normal control)

^a^: statistically significant difference with NC group

^b^: statistically significant difference with DC group

### The effects of treatments on TW

TW of untreated diabetic rats was significantly lower than those of healthy rats (*P* < 0.05) and the treatment with HESS and glibenclamide increased the weight back so that the difference was statistically significant at the highest dose of HESS and glibenclamide (*P* < 0.05). In comparison with single-agent therapy, the combination therapy of HESS and glibenclamide did not cause more TW gain (*P* > 0.05). One-way ANOVA did not show any significant differences in GSI between the experimental and control groups (*P* > 0.05).

### Effects of treatments on sperm parameters

**Fig. 2:**
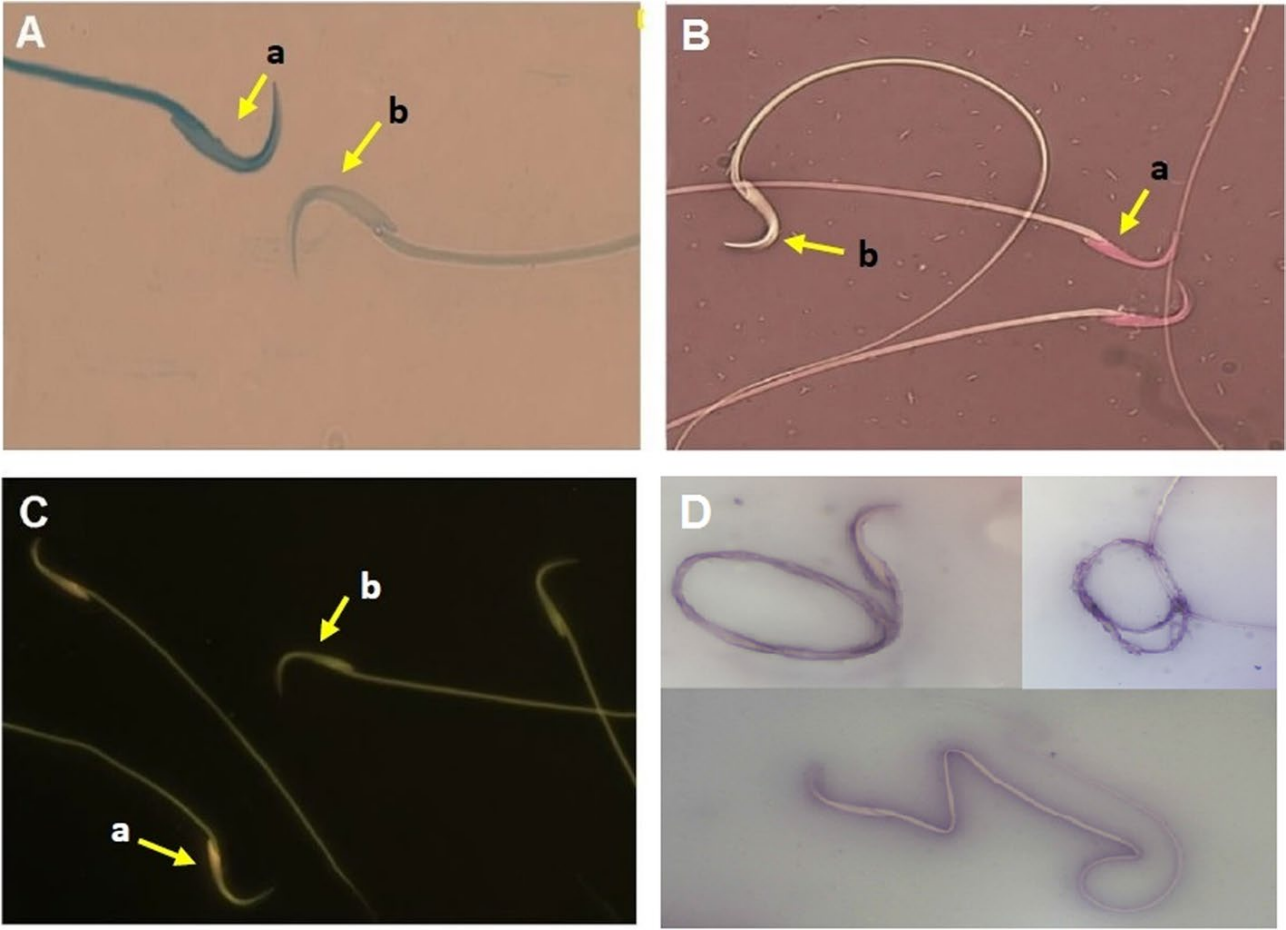
Photomicrographs of spermatozoa stained with specific methods for different purposes. A) Aniline blue for differentiation of immature (a, with a blue stained head) and mature (b, unstained) sperms. B) Eosin-Y-0.05% that distinguishes live sperms with uncolored heads (a) from dead ones with red-stained heads (b). C) Acridine-Orange, which differentiates sperms with intact chromatin structure (a, with a green fluorescence illuminating head) from those with denatured chromatin (b with a reddish-orange head. D) Eosin-Nigrosine for detection of sperms with morphological abnormalities

As shown in Table 2, the average sperm count and the percentage of mature spermatozoa (Fig. 2-A), as well as sperm motility, and viability (Fig. 2-B) were significantly reduced in diabetic rats compared to healthy control group (*P* < 0.05). HESS improved the sperm parameters dose-dependently in diabetic rats, which were significant at doses of 400 mg/kg B.W. (*P* < 0.01). Compared with the highest amount of HESS, glibenclamide more effectively (*P* < 0.01) improved the parameters to the levels detected in the healthy control group (*P* > 0.05). Combination therapy with the HESS partly enhanced the effects of glibenclamide, so that the sperm count, maturity, motility, and viability in the G + HESS-400 group were similar to those in healthy rats.

The percentages of DNA damage and sperm abnormality were determined by Acridine-Orange (Fig. 2-C) and Eosin-Nigrosine (Fig. 2-D) staining, respectively. Compared to the healthy control, both DNA damage and sperm abnormality were significantly higher in diabetic rats (*P* < 0.05). HESS treatment reduced both defects in a dose-dependent manner in the diabetic rats (*P* < 0.05), but glibenclamide was more effective than the highest dose of HESS in this regard (*P* < 0.05). Combination therapy with HESS increased the effects of glibenclamide so that the values were close to those in healthy rats (*P* > 0.05).

### Effects of HESS and glibenclamide on testicular histology

**Fig. 3:**
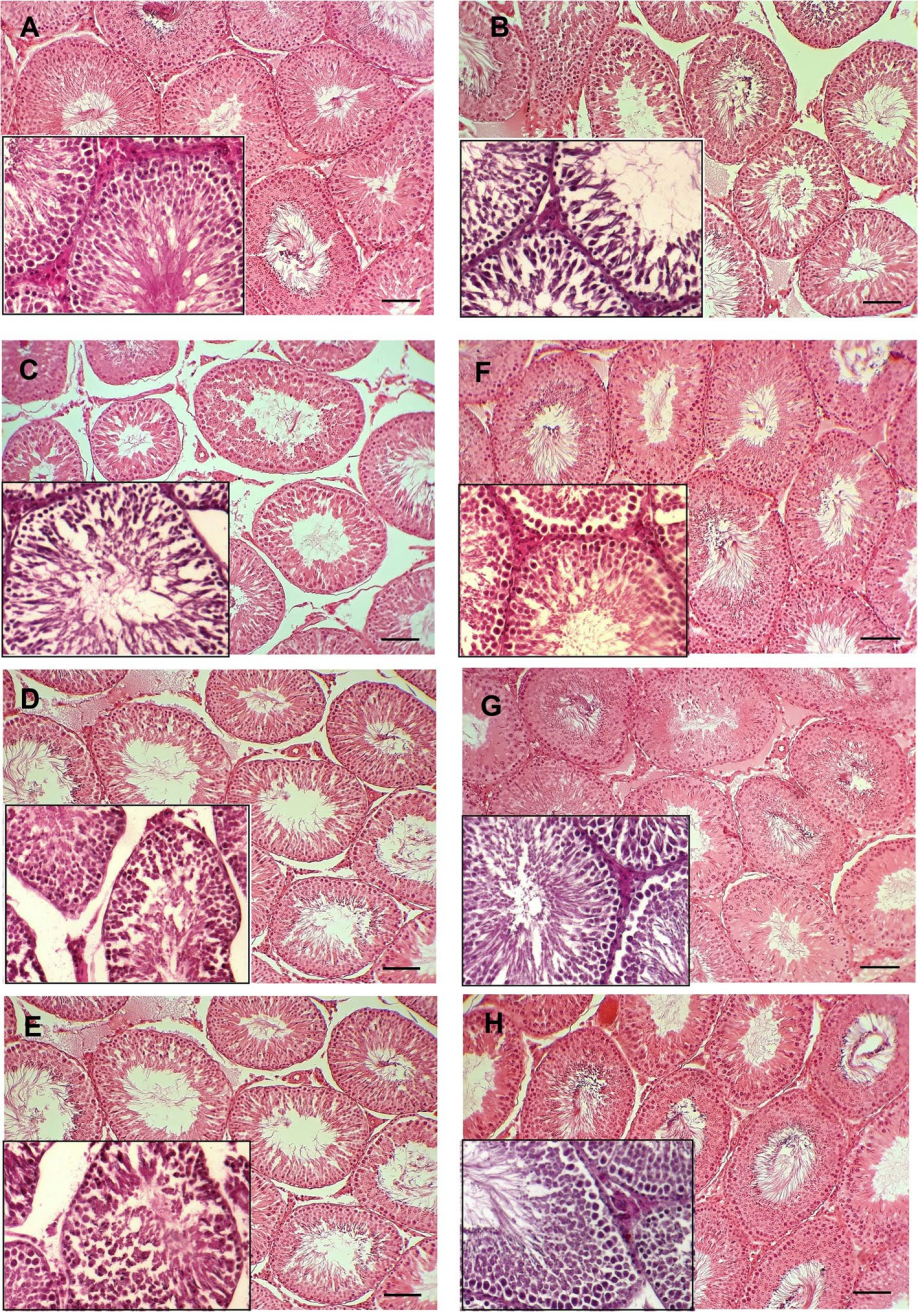
Morphological assessment of testicular tissue. **A**) Normal control group with typical seminiferous tubules showing the consecutive stages of spermatogenesis. **B**) Diabetic control group, **C**) HESS-100 group, **D**) HESS-200 group and **E**) HESS-400 group with tissue atrophy and degenerative changes in the seminiferous tubules including shrinkage, hypocellularity, vacuolation and rupture of the epithelium, and disrupted spermatogenesis. **F**) G (glibenclamide) group, **G**) G + HESS-200 group and H) G + HESS-400 group with mild histomorphological changes compared to the NC group

As shown in Fig. 3-A, the histopathological examination of testes of the NC group showed normal structure of the seminiferous tubules with typical consecutive stages of spermatogenesis, without abnormal changes in germinal epithelium and interstitial connective tissue. The hexagonal or rounded seminiferous tubules are separated with a thin intertubular interstitial connective tissue, and contain normal spermatogenic epithelium. On the contrary, the DC, HESS-100, HESS-200, and HESS-400 groups (Figs. 3-B-E) showed degenerative changes including testicular atrophy, shrunken tubules, hypocellularity, rupture, and vacuolation of the seminiferous tubular epitheliumand incomplete spermatogenesis. The spermatogenesis indices and histometrical parameters (Tables 3 and 4) were significantly deteriorated compared with the NC group. Figures 3-B-E demonstrate expansion of interstitial tissue and shrinkage of seminiferous tubuleslong with depletion of germ cells. Interestingly, all aforementioned changes in tissue characteristics of G, G + HESS-200 and G + HESS-400 groups (Figs. 3G-H), as well as the spermatogenesis indices and histometrical parameters (Tables 3 and 4) were negligible, close to the NC group.

**Table 3:** Spermatogenesis indices in the study groups

Groups	TDI (%)	SI (%)	RI (%)
NC	90.7 ± 2.5 ^b^	94.1 ± 3.0 ^b^	94 ± 2.6 ^b^
DC	60.9 ± 3.3 ^a^	49.9 ± 3.3 ^a^	55 ± 2.7 ^a^
HESS-100	64 ± 3.2 ^a^	51.1 ± 2.7 ^a^	58.2 ± 1.4 ^a^
HESS-200	68.4 ± 1.7 ^a^	57.2 ± 2.5 ^a^	59.3 ± 1.5 ^a^
HESS-400	72.8 ± 2.5 ^b^	62.9 ± 2.7 ^b^	63.7 ± 3.0 ^b^
G	81.4 ± 3.0 ^b^	78.9 ± 2.1 ^b^	82.6 ± 4.5 ^b^
G + HESS-200	88 ± 2.1 ^b^	88.8 ± 3.1 ^b^	89 ± 1.8 ^b^
G + HESS-400	89.2 ± 2.2 ^b^	90.4 ± 1.8 ^b^	89.5 ± 2.7 ^b^
P-value	< 0.001	< 0.001	< 0.001

TDI (Tubular Differentiation Index), SI (Spermatogenesis Index) and RI (Repopulation Index)

^a^: statistically significant difference with NC group

^b^: statistically significant difference with DC group

**Table 4:** Histometrical parameters in the study groups

Groups	STsD (µm)	GEH (µm)	TCD (µm)	STsLD (µm)	TDP (%)
NC	375.53 ± 17.8 ^b^	131.66 ± 13.5 ^b^	20.4 ± 2.44 ^b^	93.39 ± 6.27 ^b^	5.6 ± 1.14 ^b^
DC	204.99 ± 12.3 ^a^	85.17 ± 4.7 ^a^	39.94 ± 2.77 ^a^	67.49 ± 5.73 ^a^	16.2 ± 3.27 ^a^
HESS-100	210.91 ± 14.6 ^a^	87.75 ± 4.7 ^a^	39.72 ± 2.92 ^a^	68.92 ± 6.14 ^a^	15.8 ± 2.39 ^a^
HESS-200	218.73 ± 13.6 ^a^	89.44 ± 2.7 ^a^	36.89 ± 3.59 ^a^	68.115 ± 5.49 ^a^	15.6 ± 4.22 ^a^
HESS-400	230.1 ± 9.4 ^a^	95.9 ± 4.7 ^a^	37.91 ± 2.51 ^a^	69.6 ± 3.66 ^a^	15.6 ± 3.91 ^a^
G	319.66 ± 39.3 ^b^	122.26 ± 7.7 ^b^	26.82 ± 3.17 ^b^	84.17 ± 5.83 ^b^	8.4 ± 2.41 ^b^
G + HESS-200	339.22 ± 27.3 ^b^	127.72 ± 6.6 ^b^	22.46 ± 3.15 ^b^	87.52 ± 7.23 ^b^	7.6 ± 2.97 ^b^
G + HESS-400	356.26 ± 25.25 ^b^	129.63 ± 7.43 ^b^	22.22 ± 4.61 ^b^	89.67 ± 4.28 ^b^	6.8 ± 4.32 ^b^
P-value	< 0.001	< 0.001	< 0.001	< 0.001	< 0.001

STsD (seminiferous tubules diameter), GEH (germinal epithelium height), TCD (testicular capsule diameter), STsLD (seminiferous tubules lumen diameter), TDP (Tubular Degeneration percentage)

^a^: statistically significant difference with NC group

^b^: statistically significant difference with DC group

### Effects of HESS and glibenclamide on biochemical parameters

In this study, one-way ANOVA showed a significant difference in the measured biochemical parameters between the experimental and control groups (Table 5).

**Table 5:** Biochemical parameters of studied groups

Groups	Serum TS (ng/mL)	Tissue lactate nm/mg-tissue	Tissue LDH U/mg-total protein	Testicular glycogen ng/mg-tissue	Testicular FRAP µMol/mg-tissue	Serum insulin (ng/ml)
NC	0.098 ± 0.008^b^	3.80 ± 0.2^b^	187.1 ± 8.5^b^	0.47 ± 0.01^b^	8.66 ± 0.35^b^	1.97 ± 0.15^b^
DC	0.079 ± 0.001^a^	2.62 ± 0.29^a^	171.9 ± 1.4^a^	0.34 ± 0.02^a^	6.55 ± 0.34^a^	1.18 ± 0.09^a^
HESS-100	0.079 ± 0.001^a^	2.78 ± 0.14^a^	174.7 ± 3.0^a^	0.36 ± 0.02^a^	6.50 ± 0.36^a^	1.4 ± 0.05^a,b^
HESS-200	0.081 ± 0.004^a^	2.90 ± 0.24^a^	176.9 ± 2.9^a^	0.37 ± 0.01^a^	6.64 ± 0.37^a^	1.54 ± 0.07^a,b^
HESS-400	0.090 ± 0.003^b^	3.24 ± 0.1^a,b^	179.5 ± 1.3^b^	0.41 ± 0.08^a,b^	7.05 ± 0.12^a^	1.56 ± 0.09^a,b^
G	0.096 ± 0.003^b^	3.36 ± 0.23^b^	181.4 ± 2.2^b^	0.44 ± 0.02^b^	7.64 ± 0.28^a,b^	1.75 ± 0.07^a,b^
G + HESS-200	0.096 ± 0.001^b^	3.39 ± 0.16^b^	182.8 ± 1.1^b^	0.43 ± 0.02^b^	7.63 ± 0.41^a,b^	1.80 ± 0.09^b^
G + HESS-400	0.100 ± 0.006^b^	3.63 ± 0.31^b^	184.1 ± 1.0^b^	0.44 ± 0.02^b^	7.70 ± 0.33^a,b^	1.82 ± 0.09^b^
P-value	< 0.001	< 0.001	< 0.001	< 0.001	< 0.001	< 0.001

TS (Testosterone), LDH (lactate dehydrogenase), FRAP (ferric reducing antioxidant power)

^a^: statistically significant difference with NC group

^b^: statistically significant difference with DC group

Compared with the NC group, a significant decrease in serum insulin and TS levels was observed in the DC group (*P* < 0.001). Serum insulin levels were increased significantly and dose-dependently in the HESS-treated groups compared to DC group (*P* < 0.05). In this regard, glibenclamide was more effective than the highest dose of HESS and its combination with HESS restored insulin to the comparable levels in healthy rats (*P* > 0.05). The highest dose of HESS (400 mg/kg-BW) significantly increased the serum concentrations of TS in diabetic rats (*P* = 0.013), however, glibenclamide was more effective in this regard. Co-administration of glibenclamide with HESS increased the serum TS to equivalent levels in the NC group (*P* > 0.05).

As expected, the lower FRAP levels were observed in testicular tissue of diabetic control compared to healthy rats (*P* < 0.05). HESS administration, even at the highest dose, slightly increased antioxidant capacity in diabetic rats. Glibenclamide could increase FRAP level in diabetic rats (*P* < 0.05), but still at a lower level than the healthy rats (*P* > 0.05). Co-administration of glibenclamide with HESS did not improve the effects of standard drug. Regression analysis of TS on FRAP adjusted for groups and revealed a positive association (B = 0.009, *P* = 0.001).

Compared to the NC group, lower levels of glycogen, lactate, and LDH activity were detected in the testicular tissue of the DC group (*P* < 0.001). Administration of various doses of HESS slightly increased tissue levels of these parameters in diabetic rats, but the elevated levels became statistically significant at the highest dose of HESS (*P* < 0.05). Glibenclamide was slightly more effective than the highest dose of HESS, so that levels of glycogen, lactate, and LDH activity were close to those in the healthy group (*P* > 0.05). Co-administration of glibenclamide with HESS did not enhance the efficacy of glibenclamide.

### Gene expression

#### LDH

**Fig. 4:**
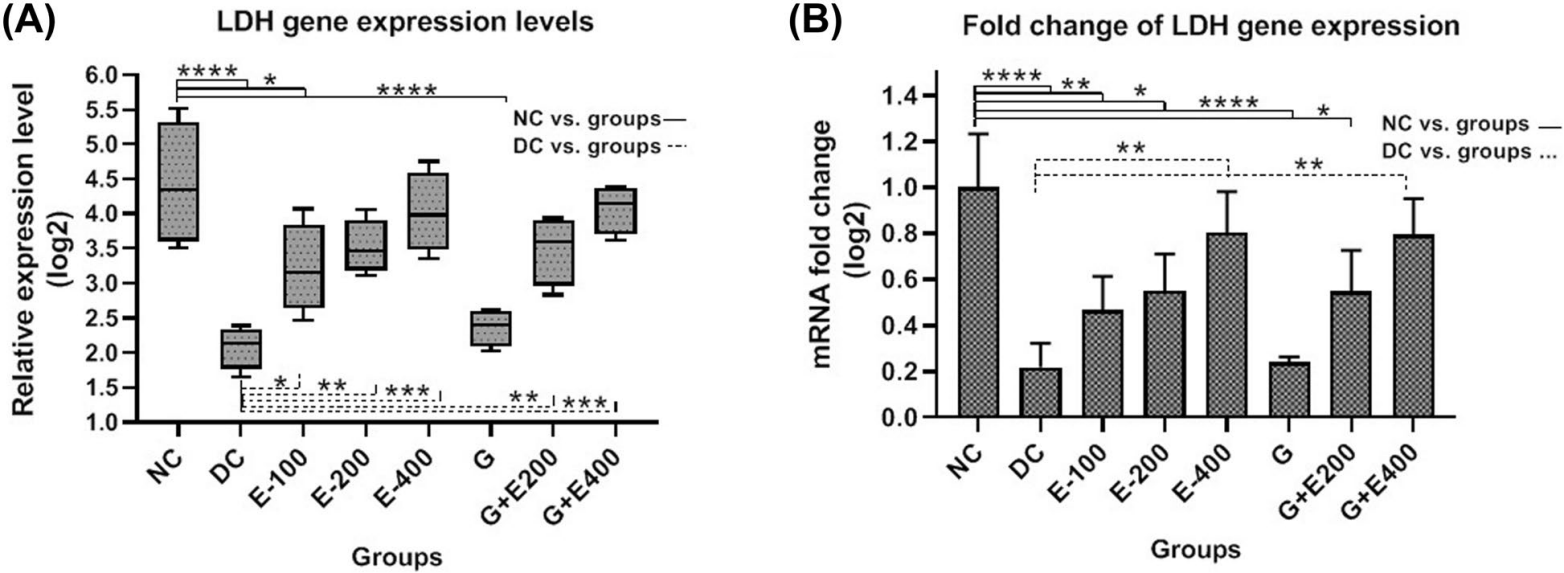
LDH gene expression levels (**A**), and comparison of fold changes (**B**) in the testicular tissue in different groups

Based on Figs. 4-A and 4-B, the LDH gene expression level in testicular tissue of healthy rats was significantly higher than that in diabetic rats (*P* < 0.05). HESS therapy increased the expression of the LDH gene in diabetic rats that were dose-dependent (*P* < 0.05). Glibenclamide was not effective in stimulating LDH expression and the increased values in G + E200 and G + E400 groups (*P* < 0.05) can be attributed to HESS as supplement.

#### FGF21

**Fig. 5:**
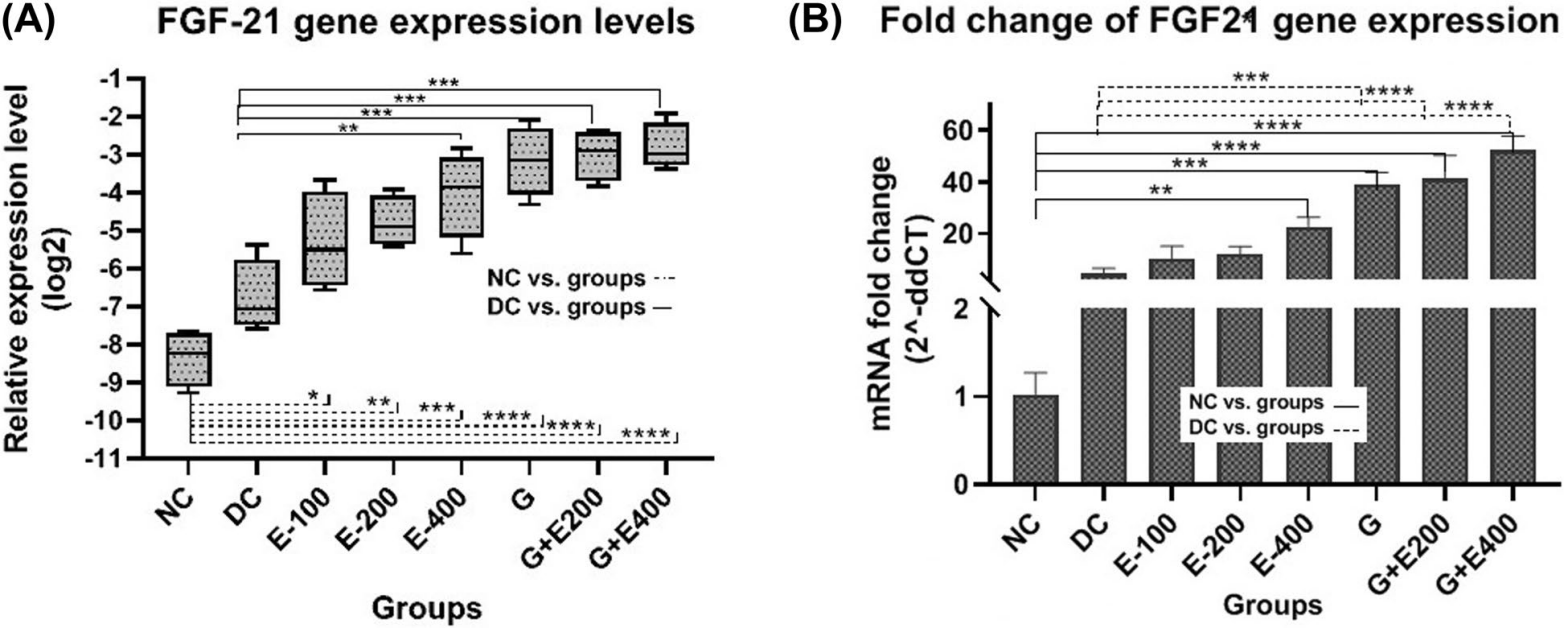
FGF21 gene expression levels (**A**), and comparison of fold changes (**B**) in the testicular tissue in different groups

According to Figs. 5-A and -B, compared to the healthy group, a mild increase in FGF21 gene expression was obseved in the testicular tissue of diabetic rats (*P* > 0.05). Treatment with HESS increased FGF21 gene expression levels dose dependantly which was significant at the highest dose of HESS compared with diabetic control (*P* < 0.05). In this regard, glibenclamide was slightly more effective than the highest dose of HESS (*P* > 0.05), and its combinational therapy with both doses of HESS (200 and 400 mg/kg.BW) did not significantly changed the FGF21 gene expression (*P* > 0.05).

Regression analysis of testicular FGF21 gene expression adjusted for FRAP revealed a positive but not significant effect of FGF-21 gene expression levels on FRAP (B = 0.02, *P* = 0.2).

## Discussion

In a previous series of studies, we examined the effects of S. securidaca, glibenclamide, and a combination of both on diabetes complications. Our previous results demonstrated the antihyperglycemic, antioxidant and antiatherogenic properties of the herbal extract, which were enhanced when combined with glibenclamide as a classical drug [24, 25]. In the current study, the side effects of S. securidaca on testicular tissue and sperm parameters in male diabetic rats were investigated and compared with the results of glibenclamide alone and in combination.

The testicular changes in diabetic animals are consistent with previous reports showing the negative effects of diabetes on intertubular tissue and fertility indices in male animals [36, 37]. The main destructive mechanisms proposed in these studies are the activation of several cellular signalling pathways by reactive oxygen species (ROS), such as the accelerated formation of AGE, the hexosamine pathway, and the polyol pathway [38, 39]. Significant testicular weight loss was observed in the testes of diabetic rats compared to normal rats. Treatment with the highest dose of HESS as well as with glibenclamide and combination of both significantly increased the testicular weight of the diabetic rats. Despite the mentioned changes in testicular mass, GSI was the same in all experimental groups and even slightly decreased in the healthy control in comparison with other groups. GSI, a parameter for assessing reproductive output, has been reported to decrease in GSI in diabetic rats [40, 41]. This discrepancy is explained by the fact that GSI is the ratio of gonad mass to the body mass, two factors that decrease in diabetes. Obviously, it is possible not to change the ratio of two numbers. Therefore, GSI parameter does not seem to be an accurate indicator in diabetes.

Histological observations of the testes of healthy control rats revealed normal hexagonal or rounded seminiferous tubules separated by a thin intertubular interstitial tissue. The normal spermatogenic layers in the germinal epithelia contained primary and secondary spermatocytes, spermatids and spermatozoa. Diabetic rats showed testicular atrophy and degenerative changes in the spermatogenic layers, associated with impaired spermatogenesis. The interstitial tissue was enlarged and the seminiferous tubules were shrunken and significantly depleted of germ cells. Thus, this study demonstrated hyperglycemia (glucotoxicity) effects on the induction of testicular tissue damage, which is due to the increased oxidative stress and cellular apoptosis in diabetes by increasing ROS production and inhibiting the antioxidant defense system [42, 43]. Treatment with different doses of HESS did not improve degenerative changes in the seminiferous tubules, but a decrease in the interstitial tissue volume with a tendency to improve seminiferous tubular structure was observed in groups treated with the highest dose of HESS, and seminiferous tubules were closer together compared to the DC group.

The observed structural abnormalities in the testicular tissues of DC, HESS-100, HESS-200 and HESS-400 groups were consistent with the estimated histometric parameters of STsD, GEH, TCD, STsLD and TDP. Compared with healthy control, the seminiferous tubule, lumen diameters and germinal epithelium height were significantly decreased, while tubular degeneration and testicular capsule diameter were significantly increased in testicular tissue of DC group. Administration of different doses of HESS improved histometric parameters in a partially dose-dependent manner; however, the changes were not statistically significant compared to the indices of the DC group. Compared with the highest administered dose of HESS, glibenclamide more effectively ameliorated the pathological conditions observed in diabetic rats. In agreement with the findings of Chatterjee et al. (2013) on the effect of glibenclamide on diabetic testis [44], regular outlines in most of seminiferous tubules and narrow intertubular interstitial tissue were observed in the sections of G group. The combination of glibenclamide with HESS normalized testicular tubules to comparable levels in healthy rats. Tissue observations were consistent with histometric parameters, in which glibenclamide alone significantly increased seminiferous tubule and lumen diameters and germinal epithelium height, while it significantly decreased tubular degeneration and testicular capsule diameter compared with the HESS-400 group. The combination with HESS partially enhanced the effects of glibenclamide on histometric parameters. Thus, glibenclamide alone was more protective than the highest dose of HESS in preventing testicular dysfunction, and the combination with HESS enhanced its effects to some extent. Similar to previous reports, diabetes had negative effects on sperm quality of rats in the group DC, such as a decrease in sperm count, motility, viability, maturity, and an increase in sperm abnormalities and DNA damage compared to healthy rats [39, 44, 45].

Histopathological examination showed that a decrease in epididymal sperm count was associated with a reduction in the number of spermatogenic cell layers in seminiferous tubules and reduced indices of spermatogenesis and tubular differentiation. Also, decrease in epididymal sperm count was a result of increase in the number of inactive spermatogonia associated with the reduction of the repopulation index. HESS dose-dependently prevented the occurrence of such sperm abnormalities in diabetic rats. Glibenclamide was more effective than the highest dose of HESS in this regard, and the combination with the highest dose of HESS improved the abnormalities to the values close to the healthy control group. The higher doses of HESS considerably prevented a decrease in sperm count and to some extent increased sperm maturation, motility and viability in diabetic rats. However, glibenclamide alone was more effective than the highest dose of HESS in this regard, and concomitant administration with the highest dose of HESS raised the sperm count, motility, viability, and percentage of sperm maturation to levels approaching those of the healthy control group. HESS prevented both DNA damage and abnormalities in the spermatozoa, but the injuries were significantly different from those in the healthy rats. Compared to the highest dose of HESS, glibenclamide more effectively prevented DNA damage and abnormalities in sperms, and the combination with HESS enhanced the effect of glibenclamide. Similar to the histometric indices, glibenclamide alone improved the spermatogenesis indices more effectively than the highest dose of HESS, and the combination with HESS improved the parameters to the values close to the healthy group. In insulin deficiency, insulin-dependent GnRH secretion is reduced, leading to decreased plasma LH and FSH levels [46]. The more significant effect of FSH on Sertoli cells in the seminiferous tubules and the greater effect of LH on Leydig interstitial cells is due to the abundance of FSH-R and LH -R in these cells. Moreover, FSH is unable to increase the number of germ cells in the absence of androgen [47]. Thus, impaired spermatogenesis and a significant decrease in serum testosterone levels are phenomena that occur in diabetes due to changes in serum LH and FSH levels. Although HESS significantly increased serum testosterone levels in diabetic rats in the present study, glibenclamide was more effective than the highest dose of HESS in this regard, and its combination with HESS normalized serum testosterone levels. In addition to hormonal action, oxidative stress, as a common phenomenon in diabetic tissue damage, interferes with the secretion of testosterone from Leydig cells [48]. Glibenclamide increased FRAP more effectively than HESS, which could be identified as another mechanism for the effectiveness of glibenclamide in improving testosterone levels in diabetic rats. Spermatogenesis is a continuous process with specific energetic requirements. As mentioned earlier, lactate produced in Sertoli cells is subsequently exported to germ cells to be used as a substrate for ATP production in mitochondria. Thus, lactate as an energy source has a crucial role in developing germ cells in the spermatogenic process [49]. It has recently been reported that glucose metabolism and lactate production are under tight hormonal control, especially by sex hormones and FSH through modulation of LDH-A, GLUT and MCT4 levels in Sertoli cells [50, 51]. Like previous studies [51, 52], this study also found a decrease in LDH gene expression and enzyme activity and, consequently, decreased testicular lactate levels in diabetic rats. Such abnormalities in the diabetic codition cause Sertoli cells to utilise their glycogen stores via the glycogenolysis pathway, which may also act as a regulator of germ cell survival during fluctuations in insulin and glucose concentrations. The observed decrease in testicular glycogen storage in STZ-treated subjects was associated with a decrease in blood insulin levels. Insulin deficiency inhibits glycogen synthesis by activating glycogen synthase kinase 3 (GSK-3), which inhibits glycogen synthase 1 (GYS1) and maintains glycogen phosphorylase in its activated form [53]. In addition, glucose uptake and lactate production by Sertoli cells are subject to hormonal regulation. Hormone receptors located in Sertoli cells are sensitive to extracellular glucose levels. Reduction in the concentration of these receptors during hyperglycemia leads to downregulation of LDH, GLUT and MCT4 in Sertoli cells [54]. Thus, glucose uptake, lactate production and its release and transport to germ cells are reduced in uncontrolled diabetes. Administration of HESS dose-dependently increased both LDH gene expression and enzymatic activity as well as lactate concentration in testicular tissue of diabetic rats. Although glibenclamide was ineffective in altering the level of LDH gene expression, it increased both LDH enzymatic activity and lactate production more effectively than the highest dose of HESS. The effect of glibenclamide on increasing LDH activity was comparable to the study by Ebokaiwe et al. [55], but we did not find studies that simultaneously assessed LDH gene expression. This discrepancy may be attributed to the possible post-transcriptional role of glibenclamide in mRNA stability, however it requires further investigation. A significant reduction in protein kinase B (AKT) phosphorylation due to insulin deficiency causes a decrease in glycogen synthase kinase-3 (GSK-3) phosphorylation and consequently an increase in glycogen synthase phosphorylation and inactivation [56]. As observed in this study, STZ-diabetic rats with lower blood insulin levels had lower testicular glycogen stores than healthy rats. The highest dose of HESS effectively increased glycogen stores in diabetic rats, which was partially similar to the effect of glibenclamide in this regard. Following an increase in blood insulin levels in the groups treated with HESS, glibenclamide, and a combination of both, testicular glycogen storage also increased. It is noteworthy that STZ at low doses (≤ 50–55) leads to incomplete destruction of pancreatic β-cells according to the previous studies [24, 57]. Treatment with higher doses of HESS, glibenclamide, or a combination of both may result in partial regeneration or repair of pancreatic β cells and insulin re-secretion from surviving β cells.

Metabolic abnormalities in diabetes increase the production of reactive oxygen/nitrogen species associated with diabetes-induced germ cell apoptosis. Nuclear factor 2 (Nrf2) is an important transcription factor that regulates the transcription of genes that have the antioxidant response element (ARE) in their promoters and encode antioxidant factors such as FGF21 [58, 59]. As mentioned before, FGF21 is a multifunctional factor produced mainly in the liver. Accordingly, it is also produced in other organs and its production is stimulated in diabetes and oxidative conditions. FGF21 maintains the homeostasis of spermatogenesis and normal germ cell apoptosis by inhibiting the activation of p53 via MDM2 through AKT [21]. The present study showed that FGF21 gene expression was higher in diabetic rats than in healthy rats, although the mean difference was not statistically significant. The highest dose of HESS significantly increased FGF21 gene expression in diabetic rats. Glibenclamide was slightly more effective than the highest dose of HESS in this regard, and its combination with HESS did not significantly change FGF21 gene expression. Basically, there are two main factors, oxidative stress and hyperglycemia, that stimulate FGF21 gene expression in diabetes. The previous studies [24, 25] indicated that the highest dose of HESS and more effectivly glibenclamid decreased blood sugar and insulin resistance, and the combination of both normalized blood sugar comparable to the healthy control group. Moreover, due to richness of HESS in flavonoids and phenolic compounds as natural antioxidants, the herbal extract was able to reduce oxidative/nitrous stress and inflammation, and slightly increased total body antioxidant capacity in a dose dependent manner [24, 25]. Morover, the highest dose of HESS slightly increased the antioxidant capacity (FRAP) of testicular tissue, glibenclamide was slightly more effective in this regard, but the combination of both did not significantly change the tissue antioxidant capacity. Here, there is a contradiction; in spite of a decrease in blood sugar, as well as oxidative stress [24] and slightly increase in tissue FRAP, the FGF21 gene expression levels significantly increased in testicular tissue of groups treated by HESS and glibenclamid. In other words, since FGF21 has been accepted as the main regulator of cell response to oxidative stress, a slight increase in tissue FRAP levels was observed despite increased FGF gene expression after treatment with HESS and glibenclamide. To justify this discrepancy through the results of previous studies, no research were found on the effect of glibenclamide as well as phenol-flavonoid compounds on FGF21 expression, and the interpretation of these results requires further research on molecular causes and cellular signaling. Jiang et al. reported that FGF21 deficiency disrupts the AKT/GSK-3/ GS and AMPK/Sirt1/PGC-1 signaling pathways in diabetic testes. This may lead to the accumulation of metabolic mediators that cause testicular oxidative damage and cell apoptosis [21]. They also showed that testicular oxidative stress was attenuated by FGF21 treatment. In another study, they reported that FGF21 deficiency activated the p53 testis in both diabetic and non-diabetic patients, which increased germ cell apoptosis [20]. Diabetes significantly inhibits testicular AKT activation and AKT-mediated glucose metabolism, which leads to impaired glucose metabolism and testicular oxidative stress. AKT regulates cell survival by modulating the activity of several apoptotic and survival proteins, including p53 [20, 60]. FGF21 increases AKT1 activation, down-regulates p53, causes a decrease in germ cell apoptosis [20]. FGF21 also modulates the metabolism of fatty acids to prevent the accumulation of their metabolic mediators, which cause oxidative stress in the testes [61]. In the present study, the positive effects of the highest dose of HESS and the stronger effects of glibenclamide on testicular tissue healing may be partly attributed to the increased tissue expression of FGF21. However, diabetes cannot be totally managed by a single or a number chemicals but by multiple chemicals and factors. Although restoration of serum insulin to the levels near normal values might be one of factors to compensate deleterious effects of diabetes, however the resone for high levels of FGF21 relation to phenolics and flavonoids as well as glibenclamid needs furder examination. Although the decrease in serum insulin to the levels near normal values might be one of the factors to compensate deleterious effects of diabetes, however, the reason for high levels of FGF21 relation to phenolics and flavonoids as well as glibenclamide needs furder examination.

## Conclusions

In a massive effort, researchers are combining standard drugs with botanicals to improve the drug's effect on diabetes and reduce disease complications such as reduced sperm quality and male infertility. This study showed that S. securidaca seeds had no side effects on testicular tissue and sperm parameters and increased sperm quality and tissue status in diabetic male rats. However glibenclamide was more effective than such a medicinal plant, its combination with the highest dose of HESS enhanced its effectiveness comparable to non-diabetics. It also facilitated to maintain the quality of sperm parameters, testicular tissue and energy homeostasis. It is concluded, *S. Securidaca* seed can be considered as an effective supplement in combination with hypoglycemic drugs to prevent reduced fertility in diabetic men.

## Data Availability

Data presented in this manuscript is available upon request.

## Abbreviations

*S. Securidaca*: *Securigera Securidaca*
HESS: Hydroalcoholic extract of *S.* *Securidaca* seed
G: Glibenclamide
NC: Normal control
DC: Diabetic control
FGF21: Fibroblast growth factor 21
LDH: Lactate dehydrogenase
MCT4: Monocarboxylate transporter 4
FRAP: Ferric reducing antioxidant power
